# Supplementary material for: Iterative improvement in the automatic modular design of robot swarms
Source: PeerJ Comput Sci. 2020 Dec 7;6:e322. doi: 10.7717/peerj-cs.322 (PMC7924708; doi:10.7717/peerj-cs.322)
Supplement: Supplemental Information 3 [file peerj-cs-06-322-s003.zip › argos3/doc/api/standalone/a00590_source.html]

ARGoS: plugins/simulator/entities/rotor\_equipped\_entity.cpp Source File


- Main Page
- Related Pages
- Namespaces
- Classes
- Files

- File List
- File Members

# plugins/simulator/entities/rotor\_equipped\_entity.cpp

Go to the documentation of this file.

```
00001 
00007 #include "rotor_equipped_entity.h"
00008 #include <argos3/core/simulator/space/space.h>
00009 
00010 namespace argos {
00011 
00012    /****************************************/
00013    /****************************************/
00014 
00015    CRotorEquippedEntity::CRotorEquippedEntity(CComposableEntity* pc_parent,
00016                                               size_t un_num_rotors) :
00017       CEntity(pc_parent),
00018       m_unNumRotors(un_num_rotors) {
00019       m_pcRotorPositions = new CVector3[m_unNumRotors];
00020       ::memset(m_pcRotorPositions, 0, m_unNumRotors * sizeof(CVector3));
00021       m_pfRotorVelocities = new Real[m_unNumRotors];
00022       ::memset(m_pfRotorVelocities, 0, m_unNumRotors * sizeof(Real));
00023       Disable();
00024    }
00025 
00026    /****************************************/
00027    /****************************************/
00028 
00029    CRotorEquippedEntity::CRotorEquippedEntity(CComposableEntity* pc_parent,
00030                                               const std::string& str_id,
00031                                               size_t un_num_rotors) :
00032       CEntity(pc_parent, str_id),
00033       m_unNumRotors(un_num_rotors) {
00034       m_pcRotorPositions = new CVector3[m_unNumRotors];
00035       ::memset(m_pcRotorPositions, 0, m_unNumRotors * sizeof(CVector3));
00036       m_pfRotorVelocities = new Real[m_unNumRotors];
00037       ::memset(m_pfRotorVelocities, 0, m_unNumRotors * sizeof(Real));
00038       Disable();
00039    }
00040    
00041    /****************************************/
00042    /****************************************/
00043 
00044    CRotorEquippedEntity::~CRotorEquippedEntity() {
00045       delete[] m_pcRotorPositions;
00046       delete[] m_pfRotorVelocities;
00047    }
00048    
00049    /****************************************/
00050    /****************************************/
00051    
00052    void CRotorEquippedEntity::Reset() {
00053       ::memset(m_pfRotorVelocities, 0, m_unNumRotors * sizeof(Real));
00054    }
00055    
00056    /****************************************/
00057    /****************************************/
00058    
00059    void CRotorEquippedEntity::SetRotor(UInt32 un_index,
00060                                        const CVector3& c_position) {
00061       if(un_index < m_unNumRotors) {
00062          m_pcRotorPositions[un_index] = c_position;
00063       }
00064       else {
00065          THROW_ARGOSEXCEPTION("CRotorEquippedEntity::SetRotor() : index " << un_index << " out of bounds (allowed [0:" << m_unNumRotors << "])");
00066       }
00067    }
00068 
00069    /****************************************/
00070    /****************************************/
00071 
00072    const CVector3& CRotorEquippedEntity::GetRotorPosition(size_t un_index) const {
00073       if(un_index < m_unNumRotors) {
00074          return m_pcRotorPositions[un_index];
00075       }
00076       else {
00077          THROW_ARGOSEXCEPTION("CRotorEquippedEntity::GetRotorPosition() : index " << un_index << " out of bounds (allowed [0:" << m_unNumRotors << "])");
00078       }
00079    }
00080    
00081    /****************************************/
00082    /****************************************/
00083    
00084    Real CRotorEquippedEntity::GetRotorVelocity(size_t un_index) const {
00085       if(un_index < m_unNumRotors) {
00086          return m_pfRotorVelocities[un_index];
00087       }
00088       else {
00089          THROW_ARGOSEXCEPTION("CRotorEquippedEntity::GetRotorVelocity() : index " << un_index << " out of bounds (allowed [0:" << m_unNumRotors << "])");
00090       }
00091    }
00092    
00093    /****************************************/
00094    /****************************************/
00095    
00096    void CRotorEquippedEntity::SetVelocities(Real* pf_velocities) {
00097       ::memcpy(m_pfRotorVelocities, pf_velocities, m_unNumRotors * sizeof(Real));
00098    }
00099 
00100    /****************************************/
00101    /****************************************/
00102 
00103    REGISTER_STANDARD_SPACE_OPERATIONS_ON_ENTITY(CRotorEquippedEntity);
00104 
00105    /****************************************/
00106    /****************************************/
00107 
00108 }
```

---

Generated on 10 Jul 2018 for ARGoS by 
 1.6.1
